# Favorable Outcome of Electively Delayed Elongation Procedure in Long-Gap Esophageal Atresia

**DOI:** 10.3389/fsurg.2021.701609

**Published:** 2021-07-06

**Authors:** Diez H. Oliver, Sidler Martin, Diez-Mendiondo I. Belkis, Wessel M. Lucas, Loff Steffan

**Affiliations:** ^1^Department of Pediatric Surgery, Klinikum Stuttgart, Stuttgart, Germany; ^2^Department of Pediatric Surgery, University Medical Centre Mannheim, Heidelberg University, Mannheim, Germany

**Keywords:** esophageal atresia, esophagoplasty, gastroesophageal reflux, anastomotic leakage, esophageal stricture, gastric pull-up

## Abstract

The ideal approach to long gap esophageal atresia is still controversial. On one hand, preserving a patient's native esophagus may require several steps and can be fraught with complications. On the other hand, most replacement procedures are irreversible and disrupt gastrointestinal physiology. The purpose of this study was to evaluate the short- and medium-term outcome of electively delayed esophageal elongation procedures before esophageal reconstruction in patients with long-gap esophageal atresia. Since the neonatal esophagus grows over-proportionally and can increase its wall thickness in the first few months of life, we hypothesized that postponing the elongation steps until 3 months of age would lead to a lower complication rate. We thus retrospectively recorded complications such as mediastinitis, anastomotic leakage, stricture formation, or gastroesophageal reflux requiring surgery, and compared it to reported outcomes. In our treatment protocol, patients born with long-gap esophageal atresia underwent gastrostomy placement and were sham fed until 3 months of age. We then assessed the gap between the esophageal ends and started serial elongation procedures. We only proceeded to the reconstruction of the esophagus when its length allowed a tension-free anastomosis. From April 2013 to April 2019, we treated 13 Patients with long-gap esophageal atresia. Nine patients without prior surgical procedures underwent Foker procedures. Four patients arrived with a pre-existing cervical esophagostomy and thus underwent Kimura's procedure, two of them with a concomitant Foker elongation of the lower pouch. Esophageal reconstruction was feasible in all patients, while none of them developed mediastinitis at any point in their treatment. We managed the only anastomotic leak conservatively. Almost half of the patients did not require any further intervention following reconstruction, while three patients required multiple (≥5) anastomotic dilatations. All but one patient achieved full oral nutrition. Only one child required a fundoplication to manage gastroesophageal reflux symptoms. Electively delayed esophageal elongation procedures in patients with long-gap esophageal atresia allowed preservation of the native esophagus in all patients. The approach had low peri-procedural morbidity, and patients enjoy favorable functional outcomes. Therefore, we suggest considering this method in the management of patients with long-gap esophageal atresia.

## Introduction

Esophageal atresia (EA) is a relatively rare congenital malformation occurring in ~1 in 4,000–2,500 live births (1, 2). In ~7% of newborns with EA, there is no fistula between the trachea and the distal esophagus. In these patients, the gap between proximal and distal esophagus is often too long for early postnatal primary repair. According to the definition of two international networks, they are hence treated as long-gap esophageal atresia (LGEA) (3, 4), although the definition of a “long gap” remains controversial (5). Moreover, the ideal surgical approach to LGEA is likely even more controversial (6), which is evident by the numerous methods of esophageal reconstruction or replacement that surgeons described since the early 20th century (7–9).

The first main option consists of esophageal anastomosis with or without prior bougienage, elongation procedure, or esophageal flap formation (10–18). Some of these methods require multiple procedures. Furthermore, complications such as tear-out of traction sutures, mediastinitis, or anastomotic stenosis are quite common in the context of traction elongation. Besides active elongation, a surgeon may elect to await over-proportional spontaneous growth of the esophagus in the first 3 months of life, and attempt a delayed primary anastomosis (DPA) (13, 19).

The second, also quite common option is esophageal replacement through gastric transposition (GT) to establish continuity between the pharynx and the stomach. Lewis Spitz published the first series of GT in LGEA in 1984 (20, 21). Although over the last three decades, GT was arguably the most common choice for esophageal replacement due to the acclaimed favorable short and medium-term outcomes compared to other forms of esophageal replacement (22–26), GT for EA has significant long-term sequelae (27).

In **our approach**, we start procedures leading to esophageal reconstruction no earlier than 3 months of age. Similar to other reports, we take advantage of spontaneous esophageal growth during the first months of life (28). Furthermore, the esophageal wall in a 3-month-old is most probably thicker and more resilient compared to the esophagus of a newborn. We aimed to verify our hypothesis that electively delaying the elongation procedures would reduce the likelihood of tearing out of traction sutures, lead to fewer episodes of mediastinitis and thus result in fewer anastomotic strictures, which in turn would allow an overall favorable functional outcome when compared to early postnatal elongation or primary GT in LGEA.

Here, we report a series of 13 patients with LGEA that we managed with a combination of Puri's suggestion namely, to allow the patient's esophagus to grow over 3 months, with the addition of subsequent elongation procedures.

## Methods

We retrospectively reviewed the charts of all patients treated for LGEA at a tertiary pediatric surgery unit from April 2013 to April 2019. We recorded patient age, gender, weight, gap length, treatment modalities, short and medium-term postoperative complications, as well as functional outcomes regarding oral nutrition.

The International Network of Esophageal Atresia (INoEA) defines LGEA as “any esophageal atresia (EA) that has no intra-abdominal air” (3); similarly, the European Reference Network for Rare Inherited Congenital.

Anomalies (ERNICA) defined LGEA as “any esophageal atresia without air in the abdomen” or “any esophageal atresia with a gap of three vertebral bodies or more” (4). We thus considered patients presenting with EA and absent distal fistula to have LGEA. In our treatment protocol for LGEA, we also included one full term baby boy with type C atresia, who was born underweight and whose postnatal gap length exceeded four vertebral bodies.

### Patient Management

Unless inserted already at the referring hospital, we placed a feeding gastrostomy during the first 48 h after admission. We postponed elongation procedures until the age of 3 months to take advantage of the over-proportional growth of the esophagus in the neonate (19). Until open surgical gap assessment, patients were sham fed to help them acquire and maintain the ability to swallow. Patients without a spit fistula needed continuous suctioning of the upper esophagus with a Replogle tube. As described by others, some children had their Replogle even as outpatients for several weeks (29). In these patients, continuous suction was ensured using an electrical suction pump initially designed for thoracic surgery (Thopaz+, Medela AG, Switzerland). Outpatients were placed on a saturation monitor while asleep; furthermore, their families received training in basic neonatal live support.

### Treatment Groups

We categorized our patients in one of the following groups depending on their treatment modality.

I. Delayed primary anastomosis (DPA)
II. DPA and Foker elongation
III. DPA and Kimura with or without Foker elongation of the lower pouch
IV. Gastric transposition

### Gap Assessment

**Figure 1:**
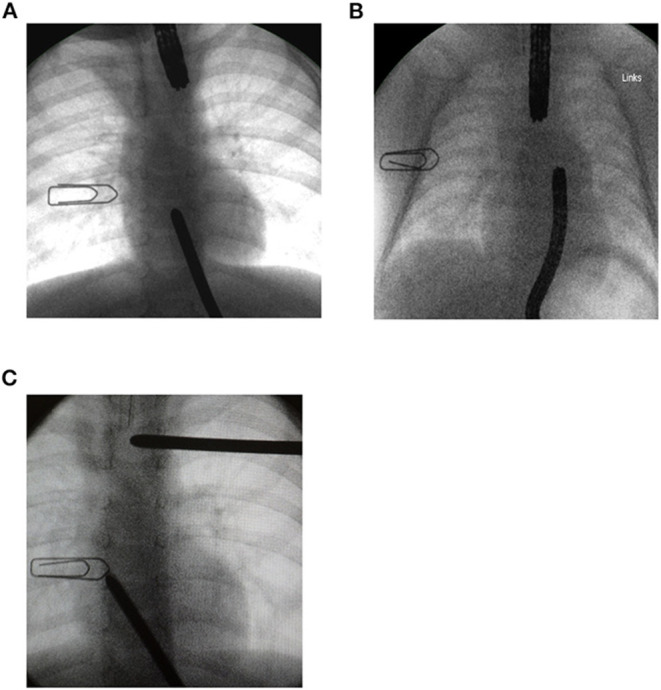
Radiographic gap assessment at 3 months of age. A 1 cm wide paperclip was placed on the patient's chest as a reference to measure gap length. **(A)** A flexible endoscope was placed in the upper pouch while a Hegar dilator marked the lower pouch. **(B)** Flexible endoscopes placed in each pouch. **(C)** One Hegar dilator placed at the site of spit fistula while the tip of the second dilator marked the upper end of the lower pouch.

We performed a gap assessment at 3 months of age or shortly after admission if they were already older than 3 months. First, non-invasive radiographic gap assessment in the absence of an esophagostomy was performed with the tip of a flexible endoscope marking the distal end of the upper part of the esophagus, while an inserted flexible endoscope or a probe marked the proximal end of the lower pouch. In patients with an esophagostomy we marked the fistula site with a radiopaque instrument. We then measured gap length with the probe or endoscope under pressure to approximate the ends of the esophagus (Figure 1). Second, 1 or 2 days following the non-invasive gap assessment, we performed an open gap assessment. The latter included open full mobilization and 15 min of gentle longitudinal traction on both pouches, or on the lower pouch only in patients with esophagostomy, respectively.

### Elongation Steps

**Figure 2:**
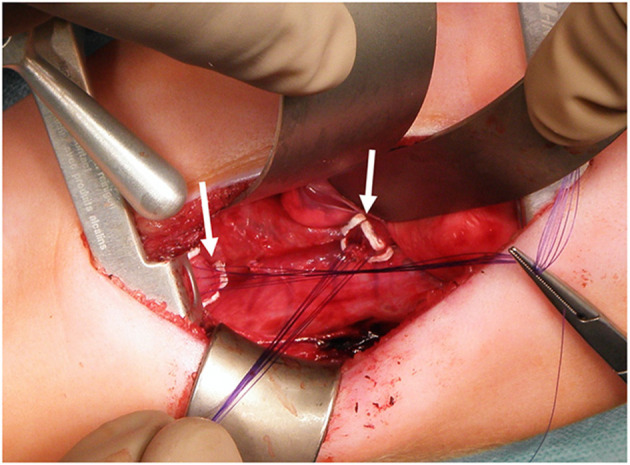
Traction sutures for Foker Procedure. We padded the sutures with PTFE-pledgets (arrows) placed on both esophageal ends to prevent tissue damage.

In patients without an esophagostomy, following the gap assessment at 3 months of age, we fully mobilized the esophageal ends and applied gentle traction for 15 min. If the ends still did not meet, we started the Foker's procedure (15, 18). We placed traction sutures as depicted in Figure 2. One week later, we reassessed gap length and either tightened the traction sutures or performed the esophageal anastomosis if the esophageal ends touched. In patients with a spit fistula, we performed Kimura elongation steps of the upper esophagus until radiographic gap assessment showed a minimal overlap of the upper and the lower esophagus (14, 16). If the gap was 4 cm or more at the initial assessment, we also performed a simultaneous Foker-type elongation of the lower pouch. All procedures were performed or supervised by the senior author. We described important technical details in the Supplemental Methods section.

## Results

From April 2013 to April 2019, thirteen patients were treated according to our LGEA protocol, while 55 patients with Gross type C EA/TEF underwent primary anastomosis. Besides these 68 patients, two patients referred to us were unsuitable for an elongation procedure and thus underwent gastric transposition; one of them suffered from an intractable long-segment esophageal stenosis following a caustic injury, while the second presented with severe complications following a failed EA-repair. Since these patients (treatment group IV) represent a very different entity, we did not include them in this report.

**Figure 3:**
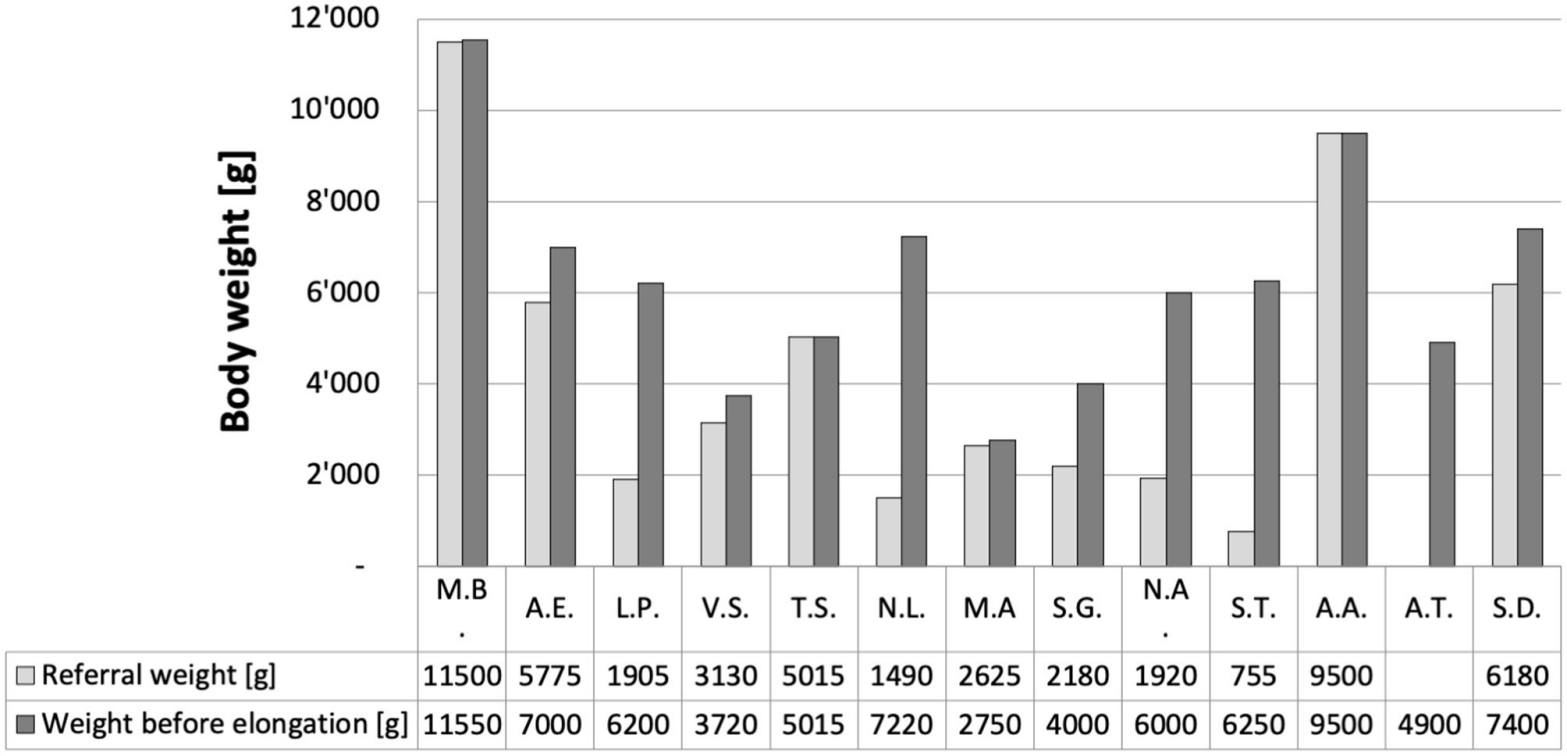
Body weight of our 13 patients. Weight at referral ranged from 755 to 11'500 g (median 2*877 g). Weight immediately before the first elongation ranged from 2'705 to 9'500 g (median 6'100 g).

The most important results were that following a median of 2 elongation steps over a median of 12 days (range 0–4 steps, or 0–249 days, respectively), tension-free anastomosis was feasible in all 13 patients. One patient referred at almost 2 years of age (M.B.) did not require an elongation. Remarkably, 12 of our 13 patients are thriving well on exclusively oral feeds, while only one patient, who was born at 25 weeks of gestation, still needed supplemental feeds through his gastrostomy at 20 months of age. At least 7 of the 13 patients were born prematurely (Table 1). Median body weight at referral was 2,877 g (range 755–11,500 g; Figure 3); median body weight at the time of the initial open gap assessment and first elongation step was 6,200 g (range 2,705–11,500 g). Median weight gain from referral to the first elongation was 1,225 g. Median radiographic gap length at no <3 months of age was 3.5 cm (range 2.1–7 cm; Table 1). Notably, the median absolute difference between radiographic gap assessment and intraoperative direct visual measurement was 2.4 cm (range 0–4 cm).

**Table 1:** Patient characteristics and features describing the course of the elongation procedures.

	**Patient characteristics**					**Treatment group**	**Elongation features**					
**Patient initials**	**Sex**	**Atresia type (Gross)**	**Spit Fistula formed at referring Hospital**	**Gestational age (weeks)**	**Radiographic gap length before elongation (cm)**		**Intraoperative gap length – after mobilization, before first elongation (cm)**	**Type of elongation**	**Number of elongation steps**	**Duration of elongation (d)**	**Loss of traction sutures**	**Mediastinitis**
M.B.	F	A	No	n/a	4	I	0	n/a	0	n/a	n/a	No
A.E.	M	A	No	n/a	3.5	II	5.5	Foker	1	7	No	No
L.P.	M	B	No	36 + 3	2.5	II	6	Foker	3	24	No	No
V.S.	F	A	No	31 + 3	4	II	1	Foker	1	9	Partial	No
T.S.	F	B	No	35 + 6	3	II	2.9	Foker	1	11	No	No
N.L.	M	A	No	32 + 0	4.7	II	5	Foker	1	5	No	No
M.A.	M	C	No	38 + 1	n/a	II	3	Foker	4	32	No	No
S.G.	M	A	No	36 + 1	2.1	II	4.5	Foker	3	21	No	No
N.A.	M	B	No	37 + 0	n/a	II	3	Foker	2	11	No	No
S.T.	M	A	No	25 + 4	3	II	3	Foker	2	13	No	No
A.A.	M	B	Yes	n/a	5	III	n/a	Kimura; Foker lower pouch	4	249	No	No
A.T.	M	A	Yes	34 + 0	3	III	n/a	Kimura	3	116	n/a	No
S.D.	F	A	Yes	n/a	7	III	n/a	Kimura; Foker lower pouch	1	9	No	No

*Treatment Groups: (I) Delayed primary anastomosis (DPA); (II) DPA with Foker elongation; (III) DPA with Kimura elongation of the upper pouch w/ or w/o Foker elongation of the lower pouch*.

Four patients with a spit fistula were referred to us from abroad. While anastomosis was feasible in one of them after mobilization of both esophageal ends, the other three underwent Kimura procedures of the upper pouch. In two of the three patients undergoing Kimura steps of the upper esophagus, we simultaneously performed traction elongation of the lower pouch. Among the four patients with a cervical esophagostomy, three had a Gross type A (Vogt type II) EA. However, the fourth of them (A.A., referred to us from the Middle East) was initially also presumed to have a pure EA. However, persistent respiratory symptoms led us to repeat radiographic and endoscopic examinations that eventually revealed a very narrow proximal fistula. In all nine patients without a spit fistula, initial management with a Replogle tube was uneventful. Patient T.S. had a Gross type B (Vogt Type IIIa) atresia. We closed the fistula of the upper pouch via a right cervical approach on day 51 of life.

In the overall cohort, we performed a total of 21 Foker elongation steps (mostly involving both pouches) with only one partial loss of traction sutures as 2 out of 4 sutures cut through the proximal end of the lower pouch (Table 1). In one patient (A.A), the four elongation steps took 249 days before we were able to complete the anastomosis; although we suggested gastric transposition in this case, the patient's family insisted in further attempts to elongate reconstruct the child's esophagus. None of the patients developed mediastinitis at any point in their course of treatment. Only one patient had an anastomotic insufficiency, which we detected on a routine contrast study performed 10 days after esophageal reconstruction (Table 2). However, the leak healed spontaneously as expected. About half of the patients needed at least one dilatation due to anastomotic narrowing, whereas three children required five or more dilatations following the reconstruction. None of the patients required resection of an anastomotic stenosis or stent insertion. In one of the 13 patients, a fundoplication was necessary to manage gastroesophageal reflux disease. Median follow-up was 43 months (range 15–81 months; one patient lost to follow up).

**Table 2:** Postoperative complications and functional outcome.

		**Complications**				
**Patient initials**	**Treatment group**	**Anastomotic insufficiency**	**Anastomotic stenosis requiring dilatation**	**≥ 5 balloon dilatations until last follow-up**	**Fundoplication**	**Oral nutrition**
M.B.	I	No	Yes	No	No	Full
A.E.	II	No	Yes	Yes	No	Full
L.P.	II	No	Yes	Yes	No	Full
V.S.	II	No	No	No	No	Full
T.S.	II	No	Yes	No	No	Full
N.L.	II	No	Yes	No	No	Full
M.A.	II	No	No	No	No	Full
S.G.	II	Yes	No	No	No	Full
N.A.	II	No	No	No	No	Full
S.T.	II	No	No	No	Yes	Partial
A.A.	III	No	Yes	Yes	No	Full
A.T.	III	No	No	No	No	Full
S.D.	III	No	Yes	No	No	Full

*Treatment Groups: (I) Delayed primary anastomosis (DPA); (II) DPA with Foker elongation; (III) DPA with Kimura elongation of the upper pouch w/ or w/o Foker elongation of the lower pouch*.

## Discussion

In this study, we found a high proportion of LGEA to be amenable to esophageal reconstruction, along with a very low complication rate and good functional outcome. Reconstruction of the esophagus is facilitated by awaiting its spontaneous growth in the first few months of life. Furthermore, patiently repeated elongation steps, combining Foker and Kimura procedures where required, ultimately resulted in sufficient esophageal length to allow a tension-free anastomosis in all our patients (14–16, 18). Our experience is in contrast to reports where half to almost all patients with LGEA needed esophageal replacement (5, 30).

There are debatable reasons to avoid gastric transposition (GT) and to preserve the patient's native esophagus, such as maintaining the gastric reservoir function and keeping the stomach at its physiologic place (31). Esophageal reconstruction also safeguards surgical options to treat gastro-esophageal reflux disease and does not further jeopardize vagal innervation of the upper gastrointestinal tract. Additionally, esophageal replacements are not only prone to yield concerning long-term outcomes (5, 27, 32, 33), their early postoperative complications can also be severe. Moreover, failure of a replacement procedure often leaves very few options to reconstruct a form of oro-intestinal continuity.

With the herein proposed approach, we also found a low complication rate regarding the tear-out of traction sutures or anastomotic insufficiencies. Placing the Foker traction sutures in a strictly extraluminal fashion yet including sufficient esophageal tissue to prevent the sutures from cutting through can be challenging. However, this pivotal step is likely easier to perform after the esophageal wall has grown thicker at the age of 3 months compared to the neonatal period. Furthermore, in our experience, the esophageal tissue becomes much more resilient to mechanical strain over the first few months of life, which might be related to the reflux of gastric feedings into the lower esophageal stump. The improved esophageal tissue quality after the first 3 months of life most probably contributes to the low rate of anastomotic leaks in our series. Additionally, a strictly tension-free anastomosis following sufficient esophageal elongation might be even more critical. Also, the repeated elongation steps inevitably compromise the segmental blood supply and might thus promote the axial perfusion; hence, dissection of a native lower esophagus might more likely lead to tissue ischemia at the anastomosis than dissection of a previously mobilized lower pouch.

Interestingly, only one of our patients required surgical treatment for gastroesophageal reflux. Literature reviews found, that one third to one half of LGEA patients had gastro-esophageal reflux disease (GERD), and the reported ratio of anti-reflux surgery following LGEA repair can be as high as 100% (34, 35). We agree with other authors who correlate the occurrence of GER with the exerted tension when forming the anastomosis (34, 36). Furthermore, hiatal dissection during mobilization of the lower pouch might promote GER and jeopardize its blood supply (29, 32, 37). Therefore, we strictly avoid hiatal dissection to leave the position of the gastric fundus and the physiologic anti-reflux mechanism undisturbed. We assume that our low rate of GERD is at least in part owed to both, strictly supradiaphragmatic dissection and tension-free anastomosis.

Almost half of our patients did not require dilatation of an anastomotic stricture, whereas stricture formation is a very likely complication following elongation procedures; some authors even report the need for multiple dilatations in all patients following the Fokker procedure (36, 38–41). We suggest that forming a tension-free anastomosis of two well-perfused esophageal ends, which reduces the risk of a leak, along with a low prevalence of GERD, contributes to our low rate of esophageal strictures; all of these factors are notorious for promoting strictures (42, 43). During the 3-month period until the esophagus has grown in length and consists of more resilient tissue is used actively by sham-feeding the patients. Sham feeds may not only stimulate esophageal growth, but also allow the babies to acquire and maintain their swallowing skills. We speculate that patients who are at home with their Replogle tube might receive fewer oral manipulations (i.e., suctioning) than inpatients on a neonatal unit; the prior group might thus be less prone to later develop oral aversion. Sham feeds remain an important component of our approach as they might contribute significantly to the high proportion of our patients that are thriving on oral nutrition only.

We observed a considerable discrepancy between radiographic and intraoperative gap assessment. First, inaccuracy in radiographic measurement is likely due to variability in pressure applied to the endoscope or probe inserted into the esophageal parts. Furthermore, a narrow esophageal lumen may limit how deep one can insert an endoscope, or distension of the lumen may shorten the esophageal pouch. Second, the results of an intraoperative gap measurement may depend on the degree of esophageal dissection, and the tension applied to both ends. Of note, whether traction should be applied before intraoperative gap measurement is also controversial (44). In our practice, we only proceeded with an anastomosis if the esophageal ends meet without tension or dissection of the lower pouch beyond the level of the diaphragm. Otherwise, we proceed with another elongation.

While the results of our series are encouraging, the approach is still time-consuming and requires several procedures. However, considering the life-long consequences of the outcome of LGEA repair, we would argue that almost any effort is warranted to achieve the best result possible. In the context of LGEA, the different periods of treatment – from care after delivery to long-term follow-up can only be managed successfully by a multidisciplinary team, which requires specialists such as neonatologists, specialist nurses, pediatric gastroenterologists, pediatric anesthetists, pediatric intensive care, and pediatric surgeons.

There are limitations of this study, such as a limited number of patients, or missing long-term outcomes for some patients, or the retrospective data collection; therefore, some information is missing, especially information regarding prior treatment at referring centers. Also, detailed physiologic data on esophageal motility or pH-/impedance-measurement would be desirable. Furthermore, comparison with other reports about the management of LGEA patients can be difficult, as alternative definitions of long-gap EA base on absolute or relative gap length, respectively (15, 18, 45–48). As in our findings, radiologic or open gap measurement can be flawed and may be examiner dependent.

In summary, the present study reports a medium-size series of LGEA patients that underwent delayed elongation procedures before esophageal reconstruction. Our method resulted in very few short and medium-term complications. It also allowed us to preserve the patient's native esophagus in all patients, and it appears to yield favorable functional outcomes. Therefore, we suggest consideration of this method in all patients with LGEA.

## Data Availability Statement

The original contributions presented in the study are included in the article/Supplementary Material, further inquiries can be directed to the corresponding author/s.

## Ethics Statement

The studies involving human participants were reviewed and approved by Ethics committee Klinikum Stuttgart. Written informed consent from the participants' legal guardian/next of kin was not required to participate in this study in accordance with the national legislation and the institutional requirements.

## Author Contributions

Surgeons who had been operating the children were LS and DO, they established the methods described. SM, D-MB, and WL were contributing with most of the writing and scientific work-up. All authors contributed to the article and approved the submitted version.

## Conflict of Interest

The authors declare that the research was conducted in the absence of any commercial or financial relationships that could be construed as a potential conflict of interest.

## Supplementary Material

Supplementary Figure 1 Final step of a Kimura procedure. The proximal esophagus (^*^) is completely mobilized in this patient following three elongation steps and splinted with a red rubber tube before insertion into the pre-vertebral tunnel. The most recent esophagostomy site (arrow head) gets closed, the scars at the previous esophagostomy sites are still visible (arrows).

Supplementary Figure 2 **(A)** Posterior view of a patient's right chest following placement of traction sutures that were passed through the chest wall. Traction sutures of the lower esophageal pouch tied extracorporeally over a silicone pledget with an additional silicone stripe underneath (^*^). Traction sutures of the upper pouch before being tied over a silicone pledget (arrow heads). **(B)** Additional silicone stripes placed under the pledget to adjust tension on the traction sutures.
