# Supplementary material for: Favorable Outcome of Electively Delayed Elongation Procedure in Long-Gap Esophageal Atresia
Source: Front Surg. 2021 Jul 6;8:701609. doi: 10.3389/fsurg.2021.701609 (PMC8290357; doi:10.3389/fsurg.2021.701609)
Supplement: Supplementary file 1 [file Data_Sheet_1.pdf]

Supplemental Methods

Kimura procedure

The Kimura procedure was carried out according to the published method(14). In brief, we excised the spit fistula, including a small rim of skin adjacent to the esophageal mucosa. We placed traction sutures before carefully mobilizing the spit fistula along its subcutaneous course and between the collar muscles, avoiding injury to the wall of the esophagus and any adjacent tissue. We then refashioned the esophagostomy approximately 2 cm lower on the chest. At intervals of 8 weeks, a 2nd, 3rd, or even 4th elongation step followed as necessary.

When performing the completion step of the Kimura-procedure, we again meticulously dissected the elongated spit fistula subcutaneously and mobilized it even further up than before towards the prevertebral fascia. In doing so, we paid particular attention to avoid damage to the laryngeal recurrent nerve (Supplemental Figure 1). Using blunt dissection, we then created an approximately 8cm long tunnel along the prevertebral fascia. Into that tunnel, we then placed the mobilized upper pouch splinted with a Charrière 10 red rubber tube before closing the collar wounds and turning the patient in a left lateral position. Upon right-sided thoracotomy, identification of the elongated spit fistula was generally straightforward. We usually had to resect about 1 cm of the proximal esophagus, since that tissue was altered and fibrotic after several mobilizations, and thus unsuitable for an esophageal anastomosis. We performed the latter in the usual fashion following dissection of the lower pouch. The surgeon needs to anticipate some loss of length of the upper esophagus when deciding between a further elongation step versus proceeding with the anastomosis.

Foker procedure

We carried out the Foker procedure guided by the initial description of the method(15). Following thoracotomy, in a left lateral position, we identified the pouches and mobilized them while carefully avoiding damage to their muscular wall. We then placed four traction sutures 5-0 PDS armed with 3 x 4 mm PTFE-pledgets at both ends of the pouch es (Supplemental Fig. 2).

Of note, the sutures should not enter the lumen of the pouches yet enclose enough wall to prevent them from pulling through. Precise placement of the traction sutures seems to be a crucial step in the procedure. In some cases, a thin silicon sheet was wrapped around each pouch to facilitate the easy gliding of the pouches. Then the traction sutures of each pouch were brought out postero-lateral the right side of the chest using a Gauge 1 needle to pass the suture through the chest wall. Following the closure of the thoracotomy, the two ends of one traction suture were individually passed through a 1 mm strong 12 x 12 mm silicone pledget and tied. In the end, the two pouches had each a corresponding silicone pledget on the skin, and we then tied the two ends of each traction suture without tension over the silicone pledget (Supplemental Fig. 2). An additional 6 x 30 mm silicone strip was inserted underneath the 12 x 12 mm pledget to place the sutures under mild tension that could then be adjusted the following days by underlaying additional silicone strips. This arrangement of silicone pledgets and stripes also protected the skin well (Supplemental Fig. 2).
