# Supplementary figures and images for: Favorable Outcome of Electively Delayed Elongation Procedure in Long-Gap Esophageal Atresia
Source: Front Surg. 2021 Jul 6;8:701609. doi: 10.3389/fsurg.2021.701609 (PMC8290357; doi:10.3389/fsurg.2021.701609)

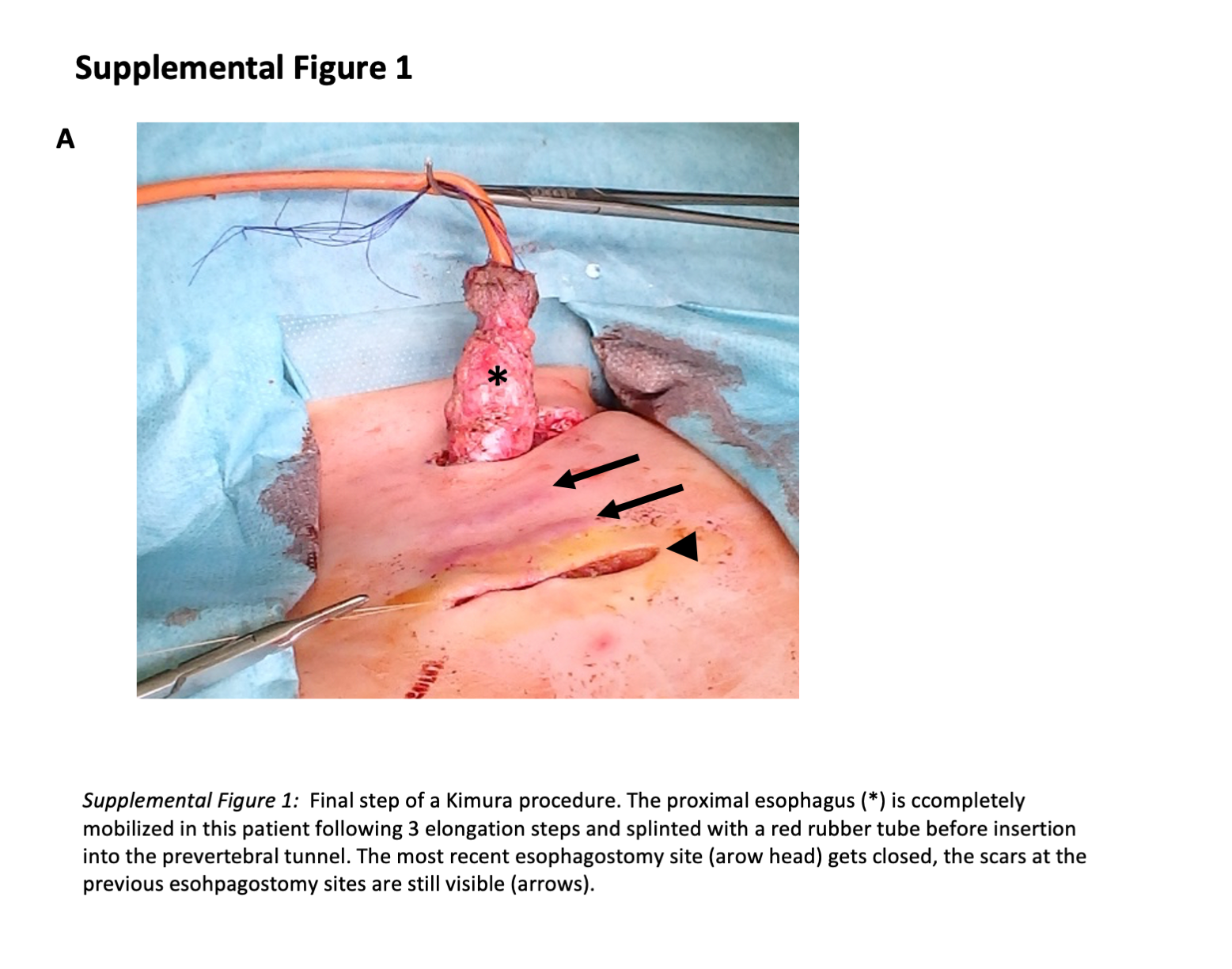

Supplement: Supplementary Figure 1 — Final step of a Kimura procedure. The proximal esophagus (*) is completely mobilized in this patient following three elongation steps and splinted with a red rubber tube before insertion into the pre-vertebral tunnel. The most recent esophagostomy site (arrow head) gets closed, the scars at the previous esophagostomy sites are still visible (arrows). [file Image_1.tiff]

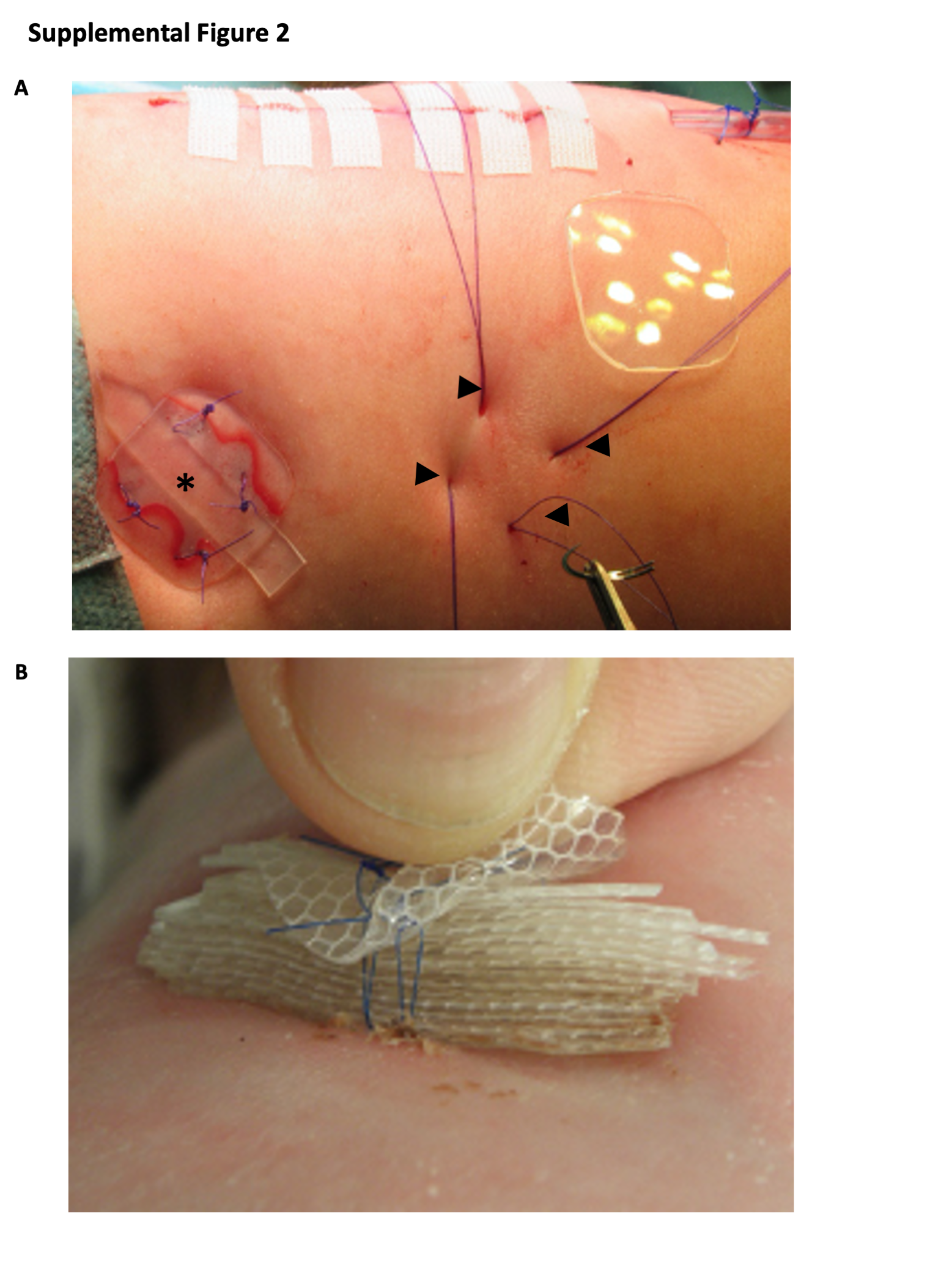

Supplement: Supplementary Figure 2 — (A) Posterior view of a patient's right chest following placement of traction sutures that were passed through the chest wall. Traction sutures of the lower esophageal pouch tied extracorporeally over a silicone pledget with an additional silicone stripe underneath (*). Traction sutures of the upper pouch before being tied over a silicone pledget (arrow heads). (B) Additional silicone stripes placed under the pledget to adjust tension on the traction sutures. [file Image_2.tiff]
